# Autocatalytic Networks at the Basis of Life’s Origin and Organization

**DOI:** 10.3390/life8040062

**Published:** 2018-12-08

**Authors:** Wim Hordijk, Mike Steel

**Affiliations:** 1 SmartAnalytiX.com, Lausanne, Switzerland; 2 Biomathematics Research Centre, University of Canterbury, Private Bag 4800, Christchurch, New Zealand; mike.steel@canterbury.ac.nz

**Keywords:** autocatalytic sets, chemical organization, RAF theory, origin of life

## Abstract

Life is more than the sum of its constituent molecules. Living systems depend on a particular chemical organization, i.e., the ways in which their constituent molecules interact and cooperate with each other through catalyzed chemical reactions. Several abstract models of minimal life, based on this idea of chemical organization and also in the context of the origin of life, were developed independently in the 1960s and 1970s. These models include hypercycles, chemotons, autopoietic systems, (M,R)-systems, and autocatalytic sets. We briefly compare these various models, and then focus more specifically on the concept of autocatalytic sets and their mathematical formalization, RAF theory. We argue that autocatalytic sets are a necessary (although not sufficient) condition for life-like behavior. We then elaborate on the suggestion that simple inorganic molecules like metals and minerals may have been the earliest catalysts in the formation of prebiotic autocatalytic sets, and how RAF theory may also be applied to systems beyond chemistry, such as ecology, economics, and cognition.

## 1. Life’s Organization

Consider the following experiment. Take some *E. coli* bacteria, put them in a petri dish with appropriate nutrients (such as glucose and some salts), and let them stand for a few days. Soon enough, the petri dish will be full of happily eating and reproducing bacteria. Now take those same *E. coli* bacteria and grind them up into their constituent molecules, place those molecules in a petri dish in a sterile environment with the same nutrients, and watch what happens. Nothing.

**Figure 1:**
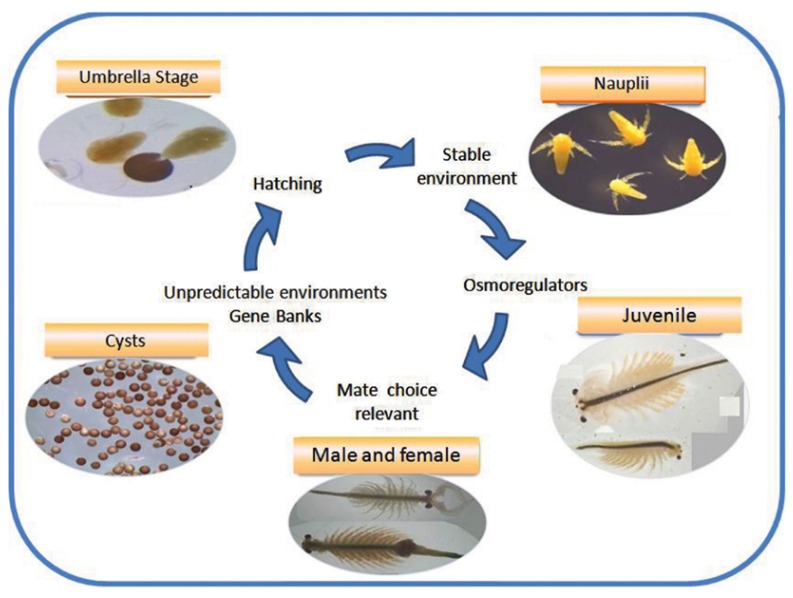
***Artemia*****life cycle.** The life cycle of the common brine shrimp *Artemia*. Figure reproduced from [2].

Next, consider an experiment that was performed more than 50 years ago [1]. Take some dried fertilized eggs from the common brine shrimp known as *Artemia* (Figure 1), store them at 2${}^{{}^{\circ}}$ Kelvin for about six days, then slowly warm them back up to room temperature, and watch what happens. The eggs hatch and the larvae grow into adults, which mate and lay eggs. In other words, after having been stored at close to absolute zero temperature for almost a week, *Artemia* continues its normal life cycle.

In both experiments, the first one with *E. coli* and the second one with *Artemia*, life was killed off. However, in the second experiment, the living state could be regenerated. What made the difference between these two experiments?

In the second experiment, with *Artemia*, life’s *organization* was not destroyed. Clearly, life is more than just the collection of its constituent molecules. There is something specific about the way in which these molecules are *organized* into a particular reaction network that gives living systems their special properties.

## 2. Formal Models

Already in the 1960s and 1970s formal models of minimal life, some also attempting to model a possible *origin* of life, were developed independently by different researchers in different places, largely based on the notion of life as an organized chemical reaction network. These models include:

- hypercycles (Eigen & Schuster, Germany),
- chemotons (Gánti, Hungary),
- autopoietic systems (Maturana & Varela, Chile),
- (M,R) systems (Rosen, USA/Canada),
- collectively autocatalytic sets (Kauffman, USA).

These models have several elements in common but they also have their differences.

Hypercycles were originally introduced as a possible way to overcome the error threshold in the template-based self-replication of biological polymers [3]. They consist of a cyclic arrangement of catalytic polymers, each one catalyzing both its own replication as well as that of the next one in the cycle. They have been studied extensively both mathematically and with computer simulations [4,5,6], and were quickly viewed as a possible mechanism in the context of the origin of life [7]. However, as far as we are aware, there are no published experimental chemical examples of hypercycles.

Chemotons were introduced as a minimal formal model of a living cell [8]. They involve three chemically coupled subsystems: (1) An autocatalytic metabolism, (2) a genetic system, and (3) a membrane. Like hypercycles, chemotons have been studied extensively both mathematically and with computer simulations [9]. However, a chemoton is generally considered to be too complex to serve as a plausible model for the *origin* of life. Furthermore, although a living cell is, by definition, an instance of a chemoton, there appear to be no published *experimental* chemical examples of chemotons.

Autopoietic systems [10] and (M,R) systems [11] are both abstract models of living systems focusing on *functional closure*. In other words, such systems produce their own components in such a way as to maintain their own internal (network) structure through which these components are produced. However, these models were formulated without any specific chemical basis. They are conceptually related [12], but have mostly remained at a highly abstract level. However, some simple autopoietic chemical systems have been constructed experimentally [13].

Finally, the concept of collectively autocatalytic sets was introduced to capture the notion of the collective replication of entire sets of molecules, which can be expected to emerge spontaneously in systems with a large enough diversity of molecule types [14]. Autocatalytic sets consist of a set of molecule types that mutually catalyze each other’s formation from a basic food source, thus focusing on *catalytic closure* as a specific (chemically-based) instance of functional closure. They were studied both mathematically and with computer simulations [15,16,17,18,19] and, contrary to most of the other models, various experimental chemical examples of autocatalytic sets do exist [20,21,22,23,24]. The notion of autocatalytic sets has been studied more extensively as RAF theory [25], which will be the focus of the remainder of this paper.

## 3. Catalysis: The Secret to Life?

A *catalyst* is a molecule that speeds up the rate at which a chemical reaction happens, without being used up in that reaction. Catalysis is ubiquitous in living systems. The majority of biological reactions are catalyzed, and catalysts are essential in determining and regulating the functionality of the chemical reaction networks that support life [26].

**Figure 2:**
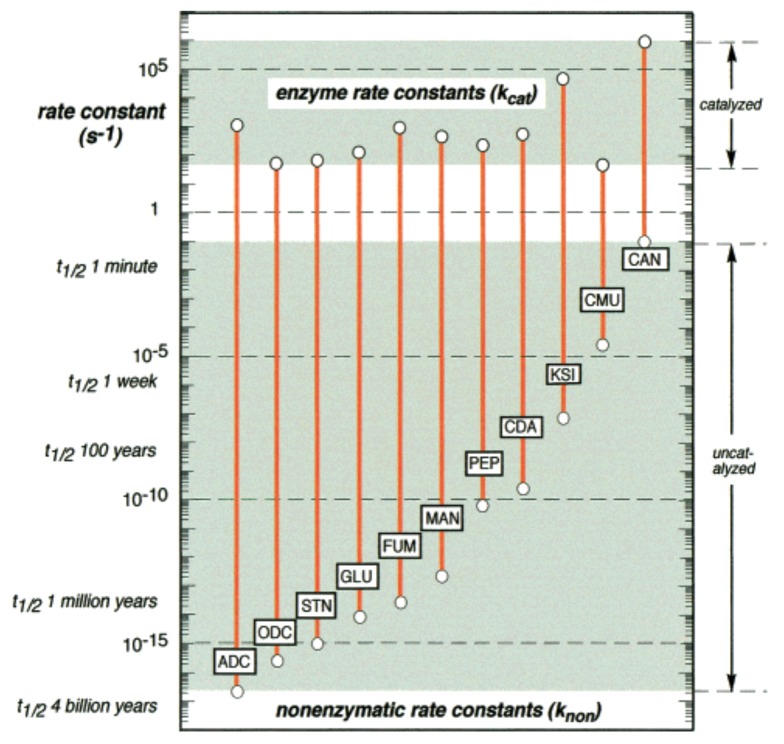
**Catalysis.** The rate enhancements of several representative reactions under the influence of enzymes. Reproduced from [27].

Not only do catalysts significantly increase the rates at which these reactions happen, but they also *synchronize* these rates more closely. For example, Figure 2 shows a comparison of uncatalyzed and (enzymatically) catalyzed rates for several biological reactions [27]. The rate increase from uncatalyzed to catalyzed reactions is many orders of magnitude. Furthermore, the *range* of rates decreases from roughly 15 orders of magnitude to only about three or four orders of magnitude. Both of these properties—an increase in the absolute rates and a decrease in the range of rates—are important for ensuring that living systems function properly [27].

In modern-day organisms, catalysis is typically carried out by highly evolved enzymes. An *enzyme* is a protein that is folded into a specific three-dimensional structure which allows it to bind efficiently to other substrates. This way, these other substrates are held in the right place so they can undergo a chemical reaction. Furthermore, this reaction is often facilitated by yet another element, often referred to as a *cofactor*, which is also held in place by the protein, and which serves as the actual catalyst. This cofactor can be a simple inorganic molecule such as a metal ion, or an organically produced molecule such as a vitamin or ATP. It is possible—perhaps even likely—that these cofactors on their own (i.e., without an encasing protein) were the original catalysts at the origin of life, a topic we will briefly return to below.

As a final remark, given the ubiquity of catalysis in living systems, it seems that life does not so much “invent” new chemistry, but rather uses chemistry that happens anyway, evolving efficient catalysts to speed up those reactions that are in some way useful for its own maintenance and reproduction. This way, a (self-)catalyzed reaction sub-network arises out of a background of all possible chemical reactions.

## 4. Autocatalytic Sets

Combining the notion of life as an organized chemical reaction network and the importance of catalysis in living systems, Kauffman introduced the concept of an autocatalytic set [14,15,16]. Simply put, an *autocatalytic set* is a set of molecules that mutually catalyze each other’s formation through chemical reactions from a basic food source. The notion of an autocatalytic set can be formalized in various ways. The one that seems the most relevant to settings such as the origin of life is the concept of a RAF (Reflexively Autocatalytic and Food-generated) set. This is a (sub)set R of reactions that simultaneously satisfies the following two conditions:

1. *Reflexively Autocatalytic* (RA): Each reaction r∈R is catalyzed by at least one molecule type that is either a product of R or is present in the food set *F*; and
2. *F-generated* (F): All reactants involved in reactions in R can be created from the food set *F* by using a series of reactions only from R itself.

**Figure 3:**
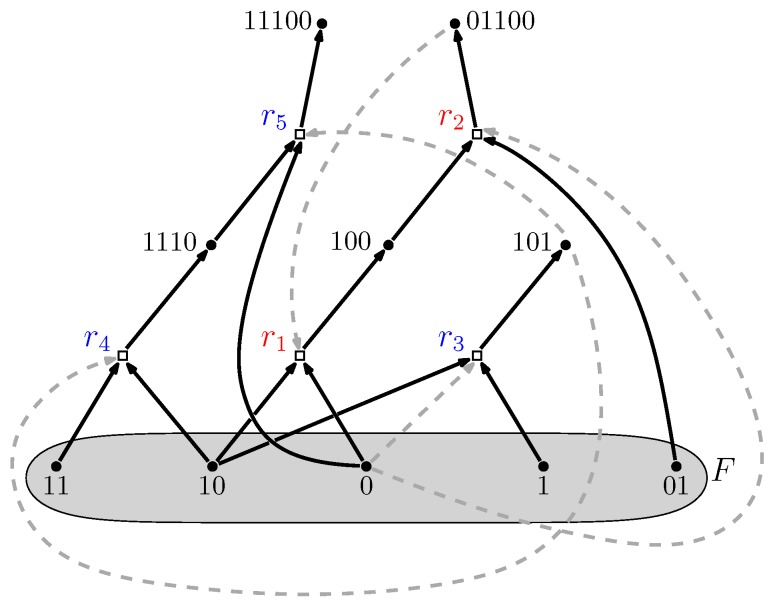
**Autocatalytic set.** An example of an autocatalytic (RAF) set that appeared in a simple polymer model where molecules are “bit string polymers” that can be ligated together into longer ones. Dots represent molecule types (labeled by bit strings); boxes represent reactions (ligations). Solid arrows indicate molecule types going into (reactants) and coming out of (products) a reaction; dashed arrows indicate which molecule types catalyze which reactions. The food set *F* consists of the monomers and dimers (i.e., bit strings of lengths one and two). Adapted from [30].

The food set *F* is a subset of molecule types that can be assumed to be available in the environment (i.e., they do not necessarily have to be produced by any of the reactions). A simple example of a Reflexively Autocatalytic and F-generated (RAF) set is shown in Figure 3. A mathematically rigorous definition of RAF sets is provided in [28,29].

An autocatalytic set thus forms a catalytically closed (RA) and self-sustaining (F) reaction network (or RAF). They have been studied extensively both mathematically and computationally as RAF theory [25]. These studies have shown that RAF sets are highly likely to exist in simple polymer models, also at realistic (and modest) levels of catalysis (defined as the average number of reactions catalyzed per molecule type) [28,31,32]. Moreover, these results hold under a wide variety of model assumptions [29,30,33,34,35,36].

Furthermore, RAF sets often consist of many hierarchical levels of subRAFs [37,38]. For example, the RAF set in Figure 3, consisting of five reactions (labeled $r_{1}$ to $r_{5}$) contains the smaller subRAFs $\left\{r_{1},r_{2}\right\}$ (indicated in red) and $\left\{r_{3},r_{4},r_{5}\right\}$ (indicated in blue). This property provides one of the necessary conditions for autocatalytic sets to be potentially evolvable [39,40,41,42]. The concept of autocatalytic sets has also been studied in related models and contexts, all giving rise to similar results in terms of the probability of their existence and potential evolvability [17,18,19,43,44,45,46,47,48,49,50,51].

However, autocatalytic sets are not just a theoretical concept. Several experimental examples have been created in the lab, either with nucleic acids or with proteins [20,21,22,23,24]. The earliest examples consisted simply of a system of two mutually catalytic nucleotide sequences, but later examples involved a set of nine peptides that mutually catalyze each other’s formation from shorter peptide fragments in various ways [22], or up to 16 ribozymes (catalytic RNA molecules) in a network of mutual catalysis [23]. Moreover, several of these experimental examples have been studied in more detail using the formal RAF framework, providing additional insights, and bringing theory and experiments closer together [52,53,54].

Note that although these experimental examples are indeed autocatalytic sets, they are not hypercycles. A hypercycle is a special (and rather restricted) instance of the more general notion of autocatalytic sets, one in which all molecule types also catalyze their own formation in addition to the formation of one or more of the other molecule types. However, in general, none of the molecule types in an autocatalytic set need to be self-replicators (although they could be).

Finally, the RAF framework has also been applied to the metabolic network of *E. coli*, showing that it forms an autocatalytic set comprising almost the entire network [55]. This brings the concept of autocatalytic sets back to the original notion of life as an organized chemical reaction network in which catalysis plays a crucial role. Indeed, an essential property of living systems is that they produce their own catalysts and, moreover, these catalysts mutually catalyze each other’s formation. This is exactly what allows living systems to evolve, diversify, and become more complex [26,56]. We therefore argue that autocatalytic sets are a *necessary* (although not *sufficient*) condition for life-like behavior.

The next section presents some more technical details of RAF theory.

## 5. RAF Theory

We refer to a catalytic reaction system (CRS) as a set of molecule types (including a food set), a set of reactions, and a pattern of catalysis (describing which molecules catalyze which reactions). Note that an arbitrary CRS does not necessarily contain a subset of reactions that forms a RAF. However, when such a RAF subset does exist, there is a unique maximal one (containing all other possible RAFs) and this unique *maxRAF* can be found by an efficient algorithm that runs in polynomial time in the size of the original CRS [28].

**Figure 4:**
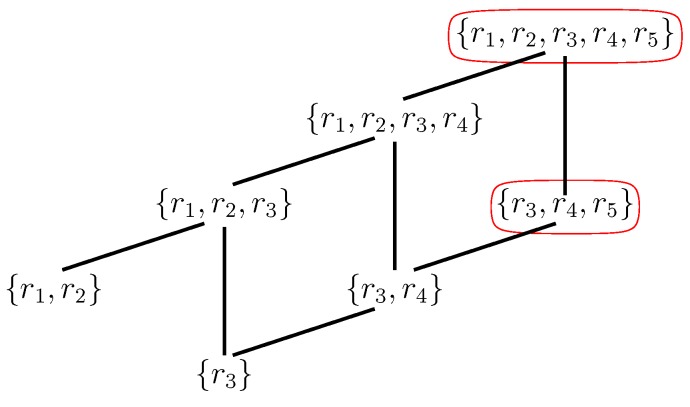
**Poset of RAFs.** The partially ordered set (poset) of all subRAFs that exist within the maxRAF of Figure 3. The maxRAF itself is at the top, while there are two irrRAFs at the bottom. The red ovals are explained in the text below.

The maxRAF together with all of its subRAFs form a partially ordered set (poset) under set inclusion. The minimal elements of this poset are called *irreducible RAFs* (irrRAFs): Removing any reaction from an irrRAF results in a set that no longer is (or contains) a RAF. In other words, they are in some sense the “smallest” RAFs, and presumably the first ones to emerge in a dynamical sense. Finding irrRAFs in any CRS can also be done in polynomial time (i.e., efficiently); however, there can be exponentially many of them (in terms of the number of reactions in the maxRAF), so enumerating all of them can, in general, cannot be done efficiently [28,30,57]. Figure 4 shows the poset of subRAFs for the maxRAF of Figure 3.

The poset of (sub)RAFs provides a formal structure to enumerate and investigate the possible ways in which small RAFs (starting with irrRAFs, at the bottom) might have evolved to larger, more complex RAFs (for example by generating more efficient catalysts, a topic we will return to below). As another example, the experimental peptide autocatalytic network described in [22] has a maxRAF of nine reactions, but it contains a total of 305 subRAFs altogether, including six irrRAFs.

However, the main subRAFs of interest, from a dynamical point of view, are those that are *closed*. This means that any reaction for which all reactants and at least one catalyst are currently available from within the subRAF will be included in it. For example, as the poset in Figure 4 shows, the subRAF $\left\{r_{3},r_{4},r_{5}\right\}$ contains an even smaller subRAF $\left\{r_{3},r_{4}\right\}$. However, this smaller subRAF is not closed: Reactions $r_{3}$ and $r_{4}$ together create the necessary reactant and catalyst for $r_{5}$, which can thus also proceed catalyzed. In other words, the subRAF $\left\{r_{3},r_{4},r_{5}\right\}$ is a closed RAF, but none of its subRAFs are. The RAF of Figure 3 contains two closed RAFs (including the maxRAF itself), which are indicated by the red ovals in the poset in Figure 4. As another example, the experimental 16-ribozyme system from [23] has only one closed RAF (namely the maxRAF). However, a simpler 7-ribozyme subsystem of this network investigated in [52] has two closed RAFs (out of 67 subRAFs in total).

A maxRAF is, by definition, always closed, but it may contain other closed RAFs within it, as the above examples show. Closed RAFs are closely connected to “organizations” in chemical organization theory (COT) [58]. Thus, the theory of chemical organizations can be used to detect and enumerate closed RAFs [59]. Although many basic questions concerning the organization of the RAF poset can be solved by efficient (polynomial-time) algorithms, some questions, such as finding (or even calculating the size of) the smallest RAF, have been shown to be NP-hard, i.e., they cannot be solved efficiently [57].

If there are closed RAFs in the poset other than the maxRAF, it implies that some reactions in the maxRAF do not immediately have all their reactants and/or catalysts present (i.e., when the system is initialized with just the food molecules). For example, in the subRAF $\left\{r_{1},r_{2}\right\}$ that is part of the maxRAF of Figure 3, reaction $r_{1}$ provides a necessary reactant for reaction $r_{2}$, which in turn provides the required catalyst for $r_{1}$. In other words, none of these two reactions can proceed catalyzed when only food molecules are present. This means that reaction $r_{1}$ will have to happen “spontaneously” (uncatalyzed) initially, before the subRAF $\left\{r_{1},r_{2}\right\}$ can come into existence dynamically.

This requirement for initial spontaneous reactions, however, provides one of the basic requirements for autocatalytic sets to be (potentially) evolvable [39,40,41,42]. Since such spontaneous reactions are rare stochastic events, different repetitions of the same experiment or simulation can give rise to different combinations of subRAFs coming into existence dynamically, potentially giving rise to different types of “protocells” that can then compete with each other (e.g., for food resources) and undergo a rudimentary form of evolution [42]. In this form of evolution, inheritance is compositional (in terms of which subRAF are currently present, dynamically), and mutations are the (spontaneous) gain or loss of a (closed) subRAF.

**Figure 5:**
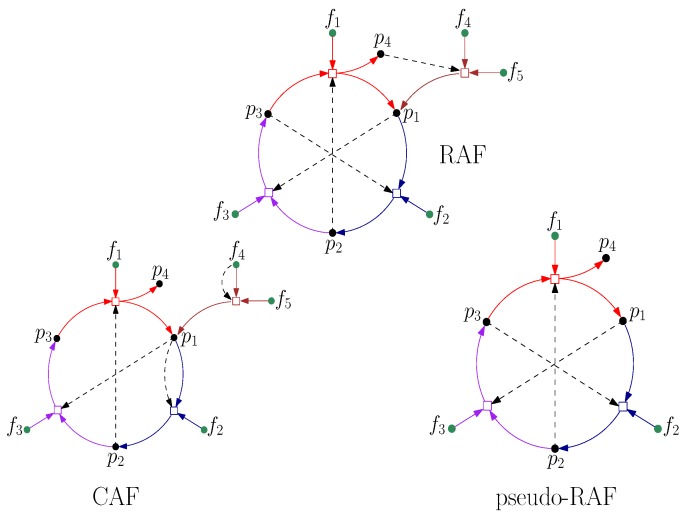
**Top:** A RAF that is not a CAF, with food set $\left\{f_{1},\ldots ,f_{5}\right\}$. Notice that several of the reactions need to happen spontaneously before all required catalysts are produced. **Bottom:** A constructively autocatalytic *F*-generated set (CAF) (**left**) and a pseudo-RAF (**right**).

A stronger concept of autocatalytic sets that requires catalysts to be already available the first time they are required leads to the more restrictive notion of a “constructively autocatalytic and food-generated” (CAF) set. CAF sets turn out to require much higher levels of catalysis to form than RAFs, and they clearly lack any compositional variety for evolution to act on (there is always only one closed CAF, namely the maxCAF). On the other hand, a notion weaker than RAFs is that of “pseudo-RAFs” (p-RAFs), in which the reactants and at least one catalyst for each reaction are produced either by some other reaction within the set (not necessarily starting from the food set) or are already present in the food set. However, this notion is also biochemically less relevant to early life than RAFs, since p-RAFs may not be food-generated (and thus not self-maintaining). These different notions are contrasted with true RAFs in Figure 5. Note that every CAF is a RAF, and every RAF is a p-RAF. The notion of a RAF thus represents a set that is able to form from a food set, yet without being too restrictive regarding the immediate availability of catalysts (i.e., some catalysts may have to be formed through reactions that are initially spontaneous and only catalyzed later).

We now turn to the probability of RAFs forming in random polymer models. For Kauffman’s original binary polymer model [16], the probability of a RAF arising undergoes a sharp transition from 0 to 1 as the expected number of reactions catalyzed by each molecule passes a certain threshold. It can be proven mathematically [32] that this threshold grows slowly (logarithmically) with the size of the CRS (and thus linearly with length of the longest polymer), and simulations show that for moderate-sized instances of the binary polymer model (involving on the order of $10^{3}$ to $10^{6}$ reactions), this critical catalysis rate requires each molecule to catalyze between one and two reactions on average. By contrast, CAFs require exponentially higher levels of catalysis [32]. An interesting feature of RAFs at catalysis rates where they are just starting to emerge in the binary polymer model is that small RAFs are highly unlikely to be present (this can be proven formally, and is also supported by simulations) [57]. However, if the catalysis rates are more heterogeneous across the molecule types, then it can be mathematically demonstrated that small RAFs also appear [36].

RAF theory has also been extended to allow for the incorporation of reaction rates, or to allow some molecule types to *inhibit* reactions [36,38,60]. Reaction rates (i.e., kinetic constants) are of course important in studying the actual dynamical behavior of RAF sets, which can be done through simulations using the standard Gillespie algorithm [61,62].

## 6. Cofactors and Coevolution

In modern-day life the chemical reactions making up an organism’s metabolism are catalyzed by highly evolved enzymes (=protein + cofactor). However, there is increasing evidence that many biological reactions can be catalyzed by inorganic elements alone [63,64,65,66]. Furthermore, many modern-day enzymes still use these inorganic elements as their cofactors [67,68].

Thus, it seems plausible that the earliest catalysts, at the origin of life, were inorganic elements that were around on the early earth anyway. These inorganic elements by themselves would likely have been much less efficient than when they are a cofactor in an enzyme, but any positive catalytic efficiency would have provided an advantage early on, compared to a background of only spontaneous (i.e., low-rate) reactions. Moreover, many of these inorganic elements would have been able to catalyze multiple reactions. Catalytic specificity probably only arose once proteins were around.

This provides a logical narrative in terms of increasingly large and complex autocatalytic sets. The first autocatalytic sets would have arisen with inorganic elements as their catalysts. Once these autocatalytic sets had established themselves, this opened up the possibility of producing more complex (organic) molecules, some of which could form more efficient catalysts, or incorporate the original inorganic elements as their cofactor making them more efficient and more specific. This, in turn, could then lead to yet more molecular complexity and catalytic efficiency, and so on until something resembling a modern-day metabolic network was formed [69]. The importance of cofactors in this process has been stressed before [55,70].

Related to this, modern life is based on two types of polymers: Nucleic acids and proteins. Both are necessary for each other’s formation. However, the standard paradigm in origin of life research, that of an RNA world, assumes that RNA arose and established itself first, with DNA and proteins relative late-comers. The standard paradigm has been questioned by many though, and one could ask whether the different types of polymers might have coevolved right from the start [71].

As mentioned above, experimental autocatalytic sets have been constructed in the lab either with RNA or with peptides [22,23,24]. It would be interesting to see if they can be made with both types of polymers together. Simulation studies have shown that in models of “partitioned” biochemical networks, where chemical reactions can happen only within one partition but catalysis can happen both within and between partitions, autocatalytic sets also have a high probability of existing, and for similar levels of catalysis as in the original (single-polymer type) models [35].

Since the formal RAF framework has already been successfully applied to the existing experimental systems, providing useful additional insights [52,53,54], such theoretical and computational studies could hopefully also serve as a guide in constructing experimental autocatalytic sets with both RNA and peptides. Furthermore, simulation studies of “partitioned” autocatalytic sets within protocells, following recent initial dynamical studies of autocatalytic sets in compartments [42], could provide additional insights into the possible early evolution of such two-polymer type systems.

## 7. Beyond Chemistry

Finally, it is possible to apply the formal RAF framework to systems beyond chemistry and the origin of life. In fact, since RAF sets are defined in a graph-theoretical way, they are not restricted to chemical reaction networks only. In principle, the nodes in a “reaction graph” could represent any kinds of entities (dots) and transformations (boxes) between those entities. For example, in modeling early life, one can regard the formation of a lipid boundary leading to an early protocell as the generation of a “higher-level” catalyst for the reactions that involve the molecules that are concentrated within the boundary (here, the catalyst is an aggregate structure rather than a single molecule) [13,72].

A more far-reaching extension was suggested recently by viewing ecosystems as a network of mutually dependent autocatalytic sets (representing species), enabling each other’s emergence and existence [73,74]. As another example, one could think of economic production functions as the equivalent of chemical reactions, where some of the produced goods in turn act as catalysts for other production functions. With this analogy in place, one could think of the economy as a whole as a catalytically closed and self-sustaining autocatalytic set [25,75]. As a final example, the RAF concept has also been applied to cognition and the origin of culture [76].

## 8. Conclusions

Already by the early 1970s, several formal models were developed (independently) based on the notion of life as an organized chemical reaction network. So far, only one of those models (autocatalytic sets) has both been studied and understood in detail mathematically and computationally and has several experimentally constructed chemical examples. Furthermore, it has been shown formally that the metabolic network of actual living organisms (or at least that of *E. coli*) can indeed be represented as an autocatalytic (RAF) set.

RAF sets have been shown to form easily in simple polymer models of chemical reaction networks, under a wide variety of model assumption, and to be, in principle, evolvable [25]. Similar computational models have shown that they are also sustainable when enclosed within small vesicles such as lipid membranes [46]. Moreover, they have been shown to form and sustain themselves in experimental laboratory settings [22,23,24].

It is plausible that the earliest catalysts in autocatalytic sets were inorganic elements such as metal ions, and that these were later incorporated as cofactors within proteins to become more efficient and specific, giving rise to larger and more complex autocatalytic sets over time. Finally, the formal RAF framework can be applied to systems beyond chemistry, such as ecosystems, the economy, and cognition. Thus, the framework represents a mathematically sound and versatile tool for studying autocatalytic sets as the basis of life’s origin and organization.

References
